# INTERSECTION OF AGE, SEX, AND FRAILTY: CONSIDERATIONS FOR TRANSLATIONAL GEROSCIENCE, GERIATRICS, AND FORTITUDE

**DOI:** 10.1093/geroni/igae098.1643

**Published:** 2024-12-31

**Authors:** Sarah Hilmer, John Mach, Susan Howlett

**Affiliations:** Chair; Co-Chair; Discussant

## Abstract

Understanding aging, optimising care of older adults and facilitating fortitude, must consider the intersection of chronological age, sex and frailty. While existing initiatives consider these factors separately, they occur together in old age and have complex interactions. This symposium brings together basic and clinical scientists, at differing career stages, from three countries, who have explored these issues in their research. Dr Alice Kane will discuss work from her laboratory in the Institute for Systems Biology, USA, on important sex differences in translational biomarkers for ageing and frailty. Dr John Mach will describe insights from a pre-clinical model of chronic drug therapy and polypharmacy, developed at the Kolling Institute, Australia, on the impact of age, sex and frailty on drug effects. Prof Sarah Hilmer, Australia, will discuss key considerations in age, sex and frailty for development of drugs for older adults, drawing on a recent position statement from the Geriatric Committee of the International Union of Basic and Clinical Pharmacologists that she chairs. Prof Heather Allore, Yale, USA, will share her expertise in biostatistics in aging research, to demonstrate design and analysis techniques that can untangle the relationship between age, sex and frailty, applicable to biological and health sciences research. Prof Paula Rochon, University of Toronto, Canada, will highlight the importance of disaggregated data on sex, age and frailty to inform research, health care and policy for older adults. Finally, Prof Susan Howlett, Dalhousie, Canada, who pioneered research in the intersection of age, sex and frailty, will lead the discussion. Women’s Issues Interest Group Sponsored Symposium

